# Using Natural Head Movements to Continually Calibrate EOG Signals

**DOI:** 10.16910/jemr.15.5.6

**Published:** 2022-12-30

**Authors:** Jason R. Nezvadovitz, Hrishikesh M. Rao

**Affiliations:** Massachusetts Institute of Technology Lincoln Laboratory, MA, USA

**Keywords:** gaze estimation, electrooculography, vestibulo-ocular reflex, sensor fusion

## Abstract

Electrooculography (EOG) is the measurement of eye movements using surface electrodes adhered around the eye. EOG systems can be designed to have an unobtrusive form-factor that is ideal for eye tracking in free-living over long durations, but the relationship between voltage and gaze direction requires frequent re-calibration as the skin-electrode impedance and retinal adaptation vary over time. Here we propose a method for automatically calibrating the EOG-gaze relationship by fusing EOG signals with gyroscopic measurements of head movement whenever the vestibulo-ocular reflex (VOR) is active. The fusion is executed as recursive inference on a hidden Markov model that accounts for all rotational degrees-of-freedom and uncertainties simultaneously. This enables continual calibration using natural eye and head movements while minimizing the impact of sensor noise. No external devices like monitors or cameras are needed. On average, our method’s gaze estimates deviate by 3.54° from those of an industry-standard desktop video-based eye tracker. Such discrepancy is on par with the latest mobile video eye trackers. Future work is focused on automatically detecting moments of VOR in free-living.

## Introduction

**Figure 1:**
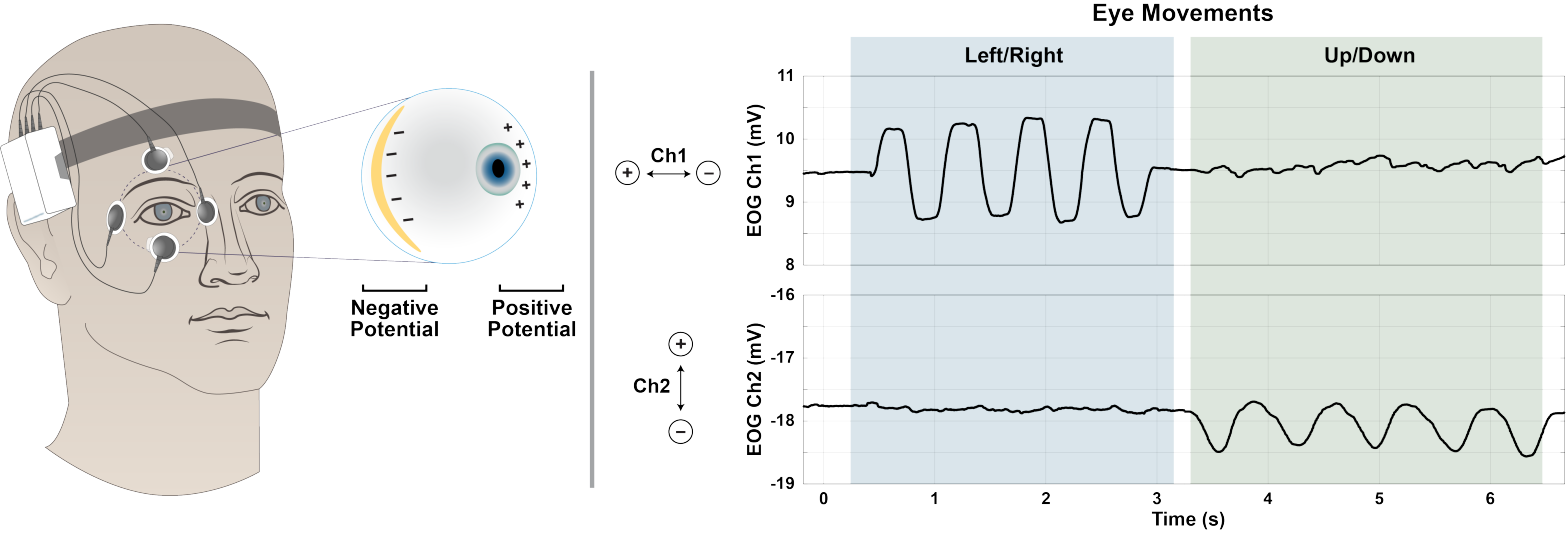
Voltage data obtained by EOG, and its relation to eye movements. Eye movements are correlated with changes in voltage measured by the surface electrodes. The electrode configuration shown was used in this work, but is not the only option.

The negatively charged retina and positively charged cornea of the human eye maintain a dipole electric field that can be measured with surface electrodes adhered to the skin around the eye. Such measurement is called electrooculography (EOG) and has established utility in the diagnosis of retinal dysfunction (29), detection of fatigue (17; 26), and human-computer interaction (4; 19). As depicted in Figure 1, the voltage measured across the electrodes varies with eye rotation. Therefore, through proper calibration, EOG can be used to estimate the orientation of the ocular axis (gaze).

The typical calibration method requires a laboratory setup where gaze direction is known by experimental construction or measured externally with a camera-based video eye tracker. The recorded EOG voltages, paired with known gaze directions, are used to fit a polynomial (20) or battery model (1). Once fit, the model can be used to estimate new gaze directions from just EOG.

However, the validity of the calibration is limited to the environmental conditions in which it was performed. Sweat alters the skin-electrode impedance, which plays a major role in the gain/scale of the measured signal (15). The lightadapted state of the retina also affects the measurement scale. In fact, the comparison of saccadic voltage amplitudes in light versus dark yields the Arden ratio, a medical diagnostic that nominally ranges from 1.7 to 4.3 (7). Therefore, in long duration data collections, the laboratory-based calibration must be repeatedly performed to maintain gaze estimate accuracy over time.

There have been many efforts to extend calibration validity by careful removal of baseline drift (2) or consideration of the statistics of saccades (14), but these techniques still rely on the constancy of a laboratory calibration of scale (be it polynomial coefficients or battery model parameters). Thus, given the inevitability of sweat and lighting changes in free-living conditions, EOGbased eye tracking has been limited to laboratories and relatively short durations between re-calibrations. This is unfortunate because EOG provides a form-factor that is ideal for long-duration use and mobile situations where other eye tracking technologies have cumbersome size, weight, and power requirements.

To enable EOG-based eye tracking in free-living conditions, the calibration process should require no external references, happen continually, and impose little to no additional burden on the user. Toward this end, we have revisited a calibration method that leverages the vestibulo-ocular reflex (VOR) – the instinctual, brainstemmediated stabilization of gaze during head movement (11). Take for example that during VOR, the gaze is fixed, so if the head rotates to the right, then the eye will counter-rotate to the left. Measurements of head movement during a VOR contain angular information about eye movement that can be used to calibrate EOG. Our proposed solution requires the simultaneous collection of EOG and head rotation measurements, but no cameras or external equipment.

The use of head rotation for VOR-based EOG calibration was first proposed in (21) and further validated in (13). However, their method requires careful decoupling of pitching (vertical) and yawing (horizontal) head rotation, thereby restricting the calibration to a lab setting in which the wearer could be instructed to make orthogonal nodding motions in sequence.

To enable calibration with *natural* head movements, we propose a hidden Markov model that accounts for all rotational degrees-of-freedom simultaneously and does not assume the exact positions of the electrodes. Furthermore, by executing the calibration as recursive Bayesian estimation, it can be run continually whenever a new moment of VOR occurs and provide a measure of uncertainty in its gaze estimates. At an algorithmic level, our approach is similar to the extended Kalman filter used for EOGbased gaze estimation in (3), but theirs does not utilize head movements for lab-free calibration.

## Methods

EOG signal calibration and 3D gaze estimation are performed jointly as recursive Bayesian estimation on a hidden Markov model. A two-channel EOG system and three-axis gyroscope affixed to the wearer’s head provide the input signals to the inference. All variables of the model are listed in Table 1. The two components of the baseline vector *b* and the two rows of the projection matrix *A* each correspond to the two channels of the EOG, though this model can easily be extended to more channels. Note that gaze is expressed as a 3D direction vector rather than a pair of angles.

**Table 1:**
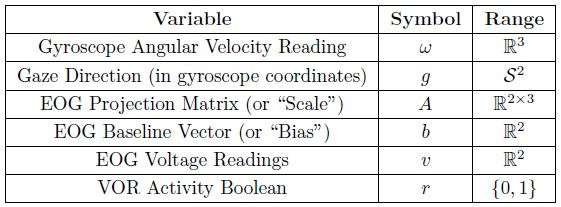
Modeled random variables. Each is a function of time and the underlying probability space. The unit-sphere S^2^ is represented as {*g* ∈ R^3^ | ⟨*g,g*⟩ = 1}. I.e., gaze is handled as a 3D direction vector rather than a pair of angles. When the wearer’s VOR is engaged, *r* is 1, else it is 0.

**Figure 2:**
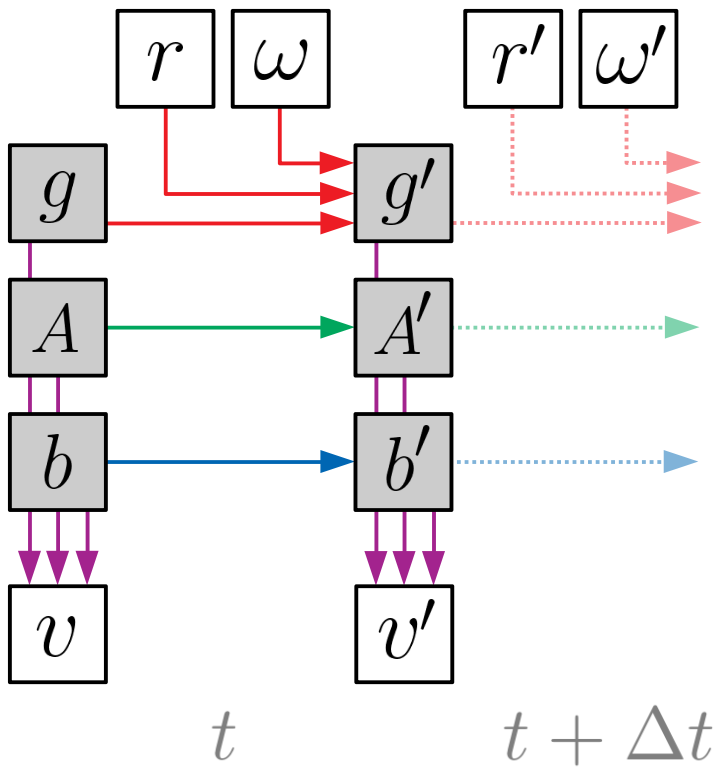
Dynamic Bayesian network for the model. Each node represents a variable and each arrow represents a conditional dependency (color-coded to match Table 2). The shaded nodes are the “hidden” states to be inferred, while the clear nodes are observed. The lighter dotted arrows indicate that this structure repeats for all timesteps (*t*+*n*∆*t* ∀*n* ∈ N). Note that the arrows connecting *g*, *A*, and *b* to *v* are passing underneath the nodes that they cross without arrowheads.

Time is discretized into small steps of duration ∆*t* ∈ R+ dictated by the gyroscope sampling rate, which, in our validation study, was 512 *Hz*. This sampling rate was selected to be high enough to capture oculomotor features; it is not a requirement of the model. The graph in Figure 2 depicts the relationships between the model variables across each time-step.

Indicated by the red arrows, the gaze rate of change (*g* to *g*^′^, i.e. eye movement) is driven by the gyroscope angular velocity readings (*ω*, i.e. head movement) and whether or not the wearer’s VOR is engaged (*r*). Indicated by the green and blue arrows, the EOG calibration coefficients (*A* and *b*) are assumed to evolve independently of the gaze and each other. This is because their drift is governed by e.g. retinal adaptation rather than gaze changes or head movement. Finally, the purple arrows indicate that the EOG voltage readings (*v*) at each time-step are specified by the gaze direction and EOG calibration coefficients at that same time-step.

**Table 2:**
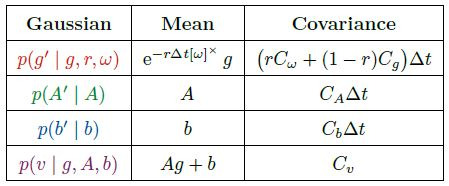
Probabilistic specification of each dependency shown graphically in Figure 2. The operator [·]^×^ expresses a 3-vector as a skew-symmetric matrix, the exponential of which is efficiently computed via the Rodrigues rotation formula.

The corresponding conditional probability distributions are all modeled as Gaussians with parameters defined in Table 2. The conditional distribution of *g*^′^ switches between two modes based on whether the wearer’s VOR is engaged or not. If *r* is 0, then the gaze evolution is modeled as a random-walk of covariance *C_g_*∆*t*, which provides a “tuning knob” to smooth-out gaze estimates by controlling how much probability is placed on large gaze changes between time-steps. If *r* is 1, then the gaze is assumed to counter-rotate the angular velocity measured by the gyroscope. The uncertainty in this relationship is encoded by the covariance *C_ω _*and is due to the gyroscope’s inherent noise, as well as the small, transient lag of the VOR (i.e., physiological latency between head and eye rotations). The trace of *C_ω _*should be much less than that of *C_g_*. Although existing research on saccadic oculomotor behavior points to non-Gaussian models (10; 24), we selected a Gaussian model with large uncertainty because it makes fewer assumptions about the nature of the non-VOR periods.

The EOG calibration parameters *A* and *b* are modeled as random-walks of covariance *C_A_*∆*t* and *C_b_*∆*t* respectively. The mean of *p*(*v* | *g,A,b*) essentially defines the role of *A* and *b* in the model: they dictate how gaze is projected / transformed into EOG readings (with noise of covariance *C_v_*). Thus the electrical properties of the eye’s dipole and the EOG (including their drift over time) are all encoded by *A* and *b*. Our results were obtained using an affine relation E[*v* | *g,A,b*] = *Ag*+*b*. However, this readily generalizes to *f*(*g*;*A*)+*b* where *f* can be any function (e.g., the battery model used in Barbara et al. [1]). Note that even when using *Ag*, the model is linear in *g* as a 3D unit-vector, not as two polar angles, so the nonlinearity of 3D dipole rotation is always captured.

**Figure 3:**
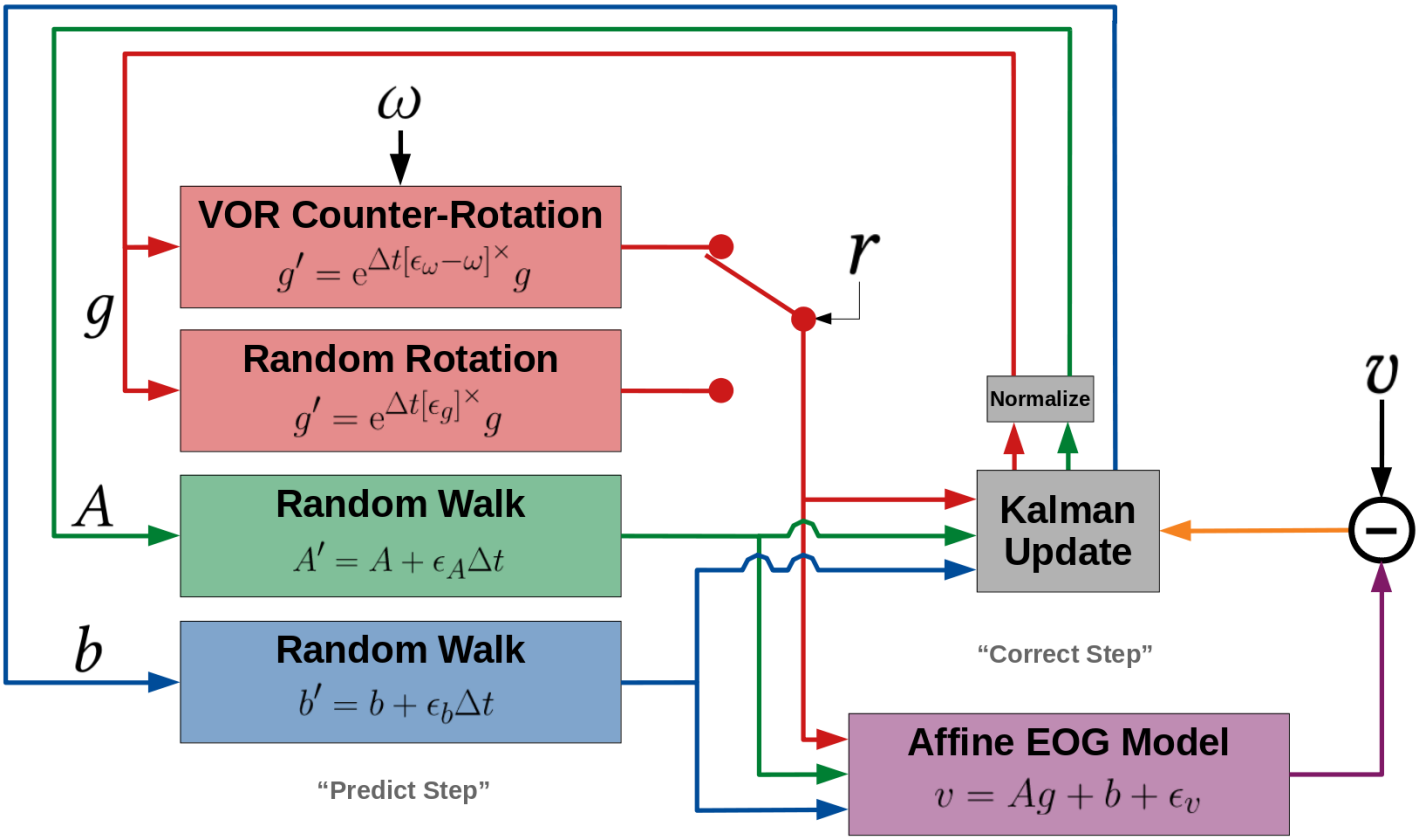
Block diagram expressing the signal path for an extended Kalman filter applied to our model. The *ϵ*_∗_ are Gaussian white-noise variables introduced to express the corresponding covariances *C*_∗_ algebraically in state-space equations.

As posed, the only full-state nonlinearity in the model is the product of *A* and *g* in E[*v* | *g,A,b*]. Thus, a nonlinear extension of the Kalman filter is advisable for performing approximate inference of the hidden states. This can be the extended Kalman filter, unscented Kalman filter, or even the Rao-Blackwellized particle filter. Satisfactory results have been obtained with the extended Kalman filter, shown schematically in Figure 3. The only non-standard piece of the implementation is renormalizing *g* after each Kalman-update by “shedding” its magnitude onto *A*. That is, to assign *A* ← |*g*|*A* and then *g* ← *g/*|*g*|. This is because the Kalman-update does not respect *g* ∈ S^2^. One could treat |*g*| = 1 as an observation with no uncertainty, but the magnitude-shedding trick exploits the *Ag* product more efficiently.

## Validation

As a proof-of-concept of the algorithm capability, a validation study was performed. Four subjects participated in the study (2 male, 2 female). All subjects provided written informed consent prior to participation. The experimental protocol was approved by the Committee on the Use of Humans as Experimental Subjects, the Institutional Review Board for MIT, as well as the Air Force Human Research Protections Office.

Our proposed methodology operates solely on raw gyroscope readings, EOG voltages, and the knowledge of when VOR is occurring. To assess the accuracy of the system, we compared the corresponding gaze estimates with those obtained by an independent, high-quality video-based eye tracker. Each trial was separated into a VOR-phase during which the calibration coefficients can be learned, followed immediately by a saccade-phase during which gaze estimation relies on what was learned during the VOR-phase. The VOR boolean *r* was set to 1 for the VOR-phase and 0 for the saccade-phase and any blinking.

During the VOR-phase, the subject fixed their gaze on a stationary target 7*m* away. It was important to use a target sufficiently far away to minimze the effect of head translation during VOR (18; 23). While maintaining fixation, the subject rotated their head in an arbitrary fashion for 30*s*.

**Figure 4:**
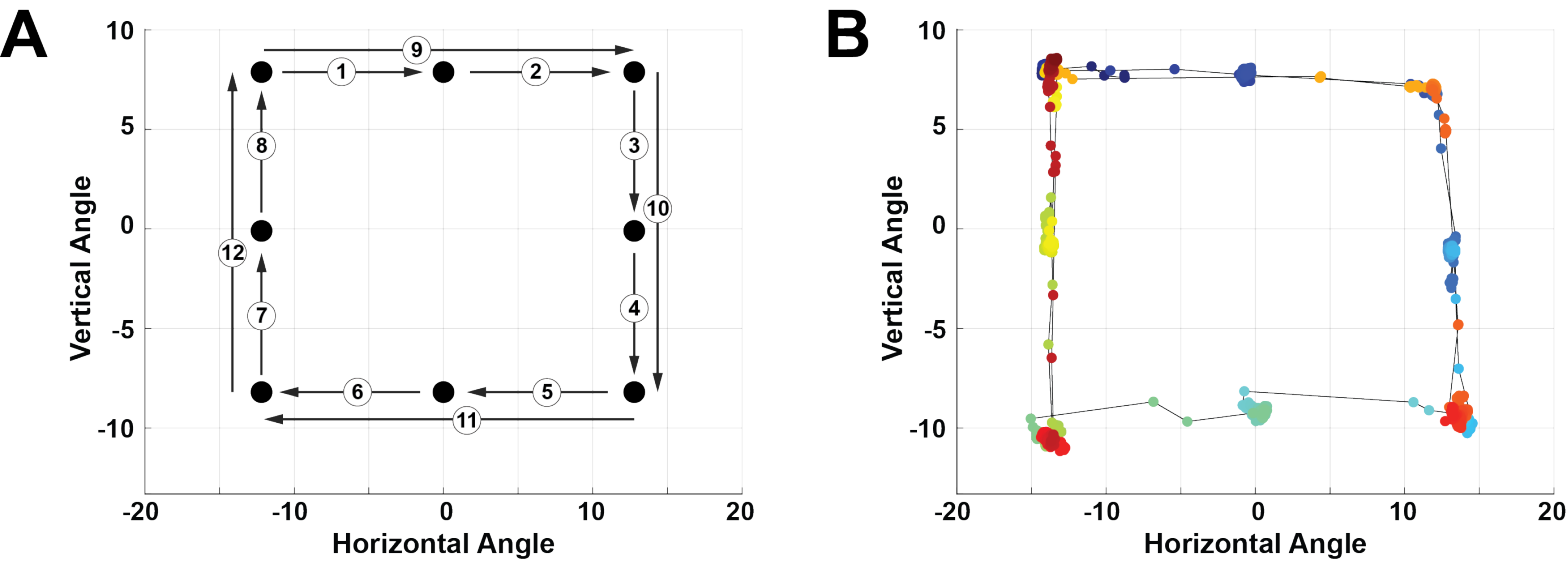
Experimental protocol for the saccade phase of each trial. **(A)** Pattern followed by the target stimulus on the screen. The numbers indicate the order of the jumps. **(B)** Data obtained by the video-based eye tracker, color-coded by time with blue being the start of the trial and red being the end.

During the saccade-phase, the subject placed their head on a chin rest roughly 60*cm* away from a computer monitor equipped with a Tobii Pro Nano video-based eye tracker (27). On the otherwise blank screen, a 0.25°-diameter circular target jumped from point-to-point in a clockwise fashion, tracing out a square. The jumps consisted of small and large jump sizes. The subject tracked the target with saccadic eye movements, and example of which is shown in Figure 4. For all subjects, while eye tracking of both eyes were collected, only data from the right eye was used.

Over both phases, our methodology was run on the EOG voltage and gyroscope data streams from a Shimmer Sensing System (Shimmer Sensing, Dublin, Ireland). The Shimmer device was affixed to an elastic headband and configured to record biopotential signals in double-differential form at 512 *Hz*. One pair of electrodes was placed above the eyebrow and below the eye, while the other pair was placed on the medial and lateral edges of the eye (see Figure 1). For all subjects, the right eye was used. The ground electrode was placed on the right mastoid bone (bony landmark behind the ear). Thus, the five-lead system yielded two channels of voltage data. Note that our model does not require nor assume this experimental configuration.

**Figure 5:**
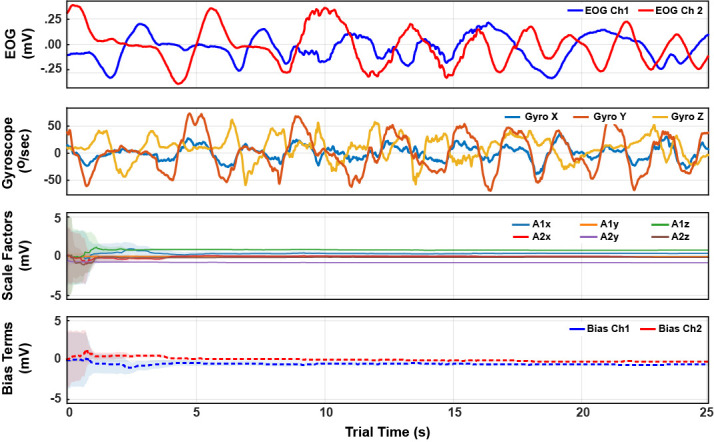
Example time series for a trial’s VOR-phase. The top two plots show the EOG and gyroscope data. The bottom two plots show the state estimate trajectories for the EOG calibration *A* and *b*. The shading indicates one standard-deviation of the inferred posterior. Within 5 seconds of arbitrary VOR motion, the posterior variances reach a small steady value, indicating convergence from arbitrary initial conditions to consistent state *tracking*.

An extended Kalman filter was used for approximate inference on the hidden Markov model. “Learning” of *A* and *b* during the VOR-phase is shown in Figure 5. The mean of *g* was arbitrarily initialized to [1*,*0*,*0]^⊺^ and the initial means of all components of *A* and *b* were drawn from N(0*,*1e−3). The state covariance was initialized to 25·*I*_11_ where *I_n _*refers to an *n* × *n* identity matrix. The following covariances were used for the model: *C_ω _*= 1e−9 · *I*_3_, *C_g _*= 1e2 · *I*_3_, *C_A _*= 1e−6 · *I*_6_, *C_b _*= 1e−2 · *I*_2_, and *C_v _*= 1e−3 · *I*_2_. They were chosen by tuning on the very first trial, and then used without any adjustment across all trials *and* subjects. The tuning did not reference the video-based eye tracker data.

**Figure 6:**
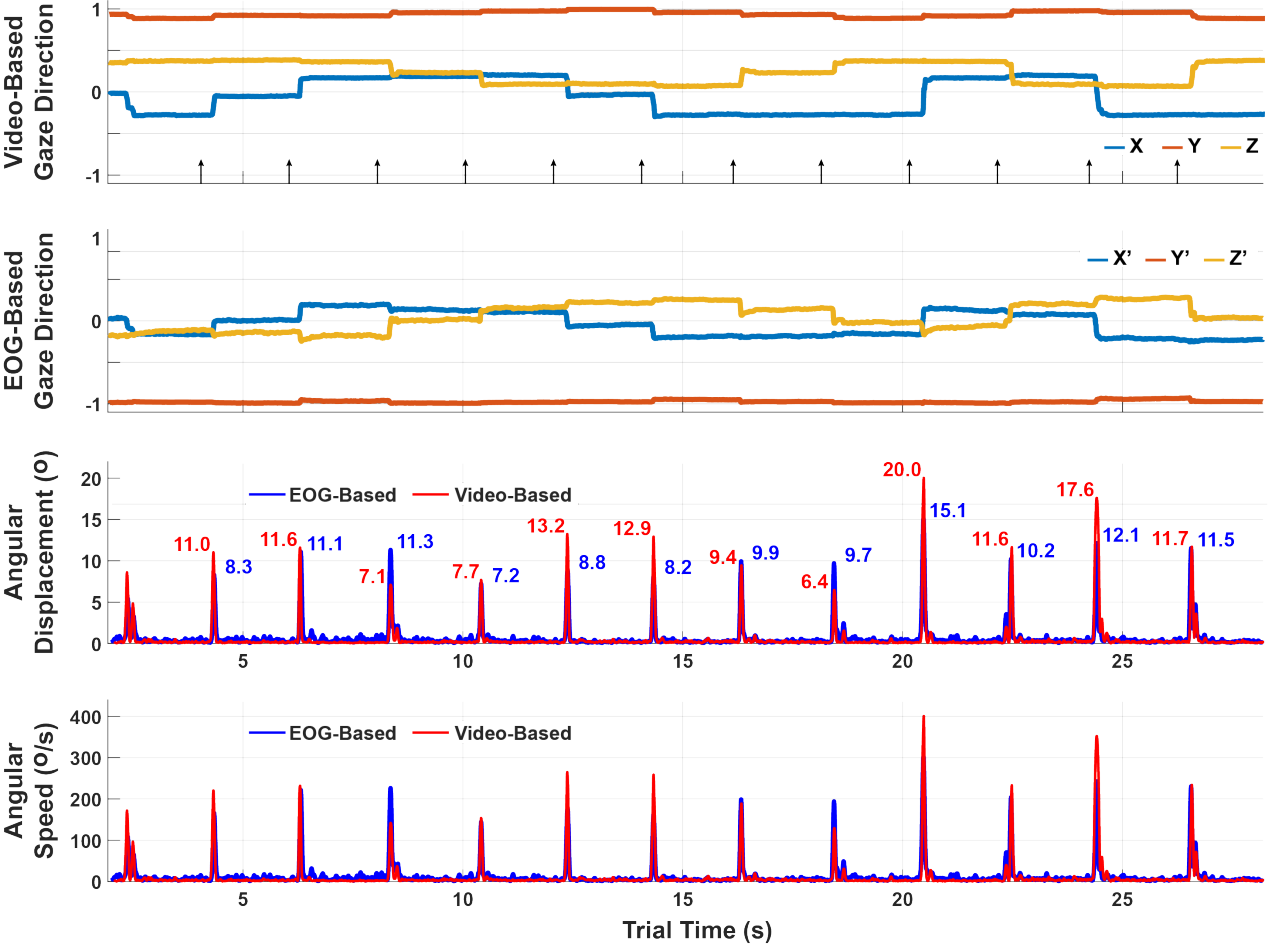
Comparison of the video-based and EOG-based gaze estimates during the saccade-phase. The top two plots show the gaze estimates themselves, which are in different coordinate systems. The bottom plots show the angular displacements and speeds over a sliding window of 50*ms*. Unlike the gaze estimates which cannot be compared component-to-component, the angular displacements are coordinate-free and can thus be compared to assess performance. The discrepancy for this trial is 2.73°±1.92° RMS between video-based and EOG-based angular displacement peaks.

The video-based and EOG-based gaze estimates are in different coordinate systems with an unknown relationship. To compare them, we transformed them into angular displacements by computing the angle between the gaze directions at every time *t* and *t* + *h* for small step-size *h*. An example is shown in Figure 6. Specifically, we define angular displacement as *δ(t)* = cos^−1^ 〈*g(t), g(t + h)*〉 and angular speed as Ω(*t*) = *δ*(*t*)*/h*, where 50*ms* was used for *h*. The saccadic eye movements made it easy to synchronize and compare the two timeseries by examining the root-mean-squared (RMS) discrepancy between the video-based and EOG-based angular displacement peaks (saccade amplitudes).

In the single trial shown in Figure 6, the RMS error is 2.73°±1.92°. The full set of errors across all subjects and trials are summarized in Table 3. The averages of the subjects are computed as the mean (± standard deviation) of errors across the trials for that subject. If the trial errors are pooled together, the average error across subjects is 3.54° ± 0.71°.

**Table 3:**
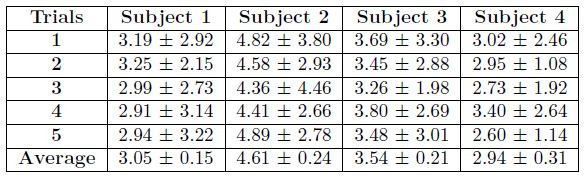
RMS (± standard deviation) discrepancies between the video-based and EOG-based angular displacement peaks during each saccade-phase. If all trials are pooled across subjects, the average discrepancy is 3.54 ± 0.71. All units are degrees.

## Discussion

The full hidden state {*g,A,b*} (gaze, scaling coefficients, and baseline) is continuously inferred through both the VOR and saccade phases, but the lack of VOR in the saccade phase decouples gaze from the gyroscope. During the saccade-phase, the eye rotation random-walk covariance *C_g _*is the dominant source of uncertainty. This causes the saccade-phase gaze estimates to essentially be derived from the *A* and *b* means at the end of the prior VOR-phase. In this regard, one can think of the inference as calibrating during VOR and then using that calibration during non-VOR (e.g., saccades and smooth pursuits). The variances of *A* and *b* grow most rapidly during non-VOR, preparing them for “recalibration” during the next VOR window.

As can be seen in Figure 5, the amount of VOR data needed to calibrate is small; the means of *A* and *b* converge after just a few seconds (hence why each trial need not exceed one minute). After this convergence, *A* and *b* continue to be tracked, thereby continually accounting for both EOG scale and baseline drift through time. In contrast, existing EOG-based eye trackers only estimate scale once at the start of a trial (via external reference like a camera) and use a low-pass filter to mitigate the effects of baseline drift, rather than explicitly estimating it through model-based inference.

The fusion of EOG and VOR information can only identify gaze direction up to an unknown but static rotational offset from anatomical coordinates. Meaning, there is no way to ensure that a gaze of say [1*,*0*,*0]^⊺^ corresponds to anatomically “looking forward” without an external reference to observe gaze in anatomical coordinates at least once. Therefore, our performance analyses have focused on angular displacements of the eye, which do not depend on choice of coordinates.

Angular displacements have merit in their own right as consistent features for physiological analysis over time and within subjects. For example, change in the saccade main sequence (the relationship between saccade speed and duration) is indicative of levels of fatigue (9), cognitive workload and attention (8), as well as clinical conditions such as traumatic brain injury (5). Nonetheless, if absolute gaze direction in anatomical coordinates is needed, it would suffice to externally measure gaze once in anatomical coordinates to determine the alignment, or to ensure that the subject is looking in a known anatomical direction (e.g. forward) upon initialization. This was not performed in the present study as the concept here is the development of a calibration method that is nonintrusive to the user.

While our current methodology treats VOR detection (*r*) as an observable / known, it is possible to extend this same framework to the case where it is another jointly inferred hidden state. However, the introduction of a binary state complicates the use of an extended Kalman filter for inference. Generally speaking, VOR detection is a matter of correlating eye movement and head movement data. While, existing literature demonstrates the capability to detect VOR using a feature-driven approach (28), caution must be taken in situations where the eye continues to move smoothly, but is not in a state of VOR.

For example, during VOR cancellation, which occurs when VOR and smooth pursuit interact (16), the eye and head continue to move smoothly, but that period should not be used for our calibration. An approach that uses just correlation between head and eye velocity may classify VOR cancellation as a window of VOR. Further, VOR detection and our calibration method may not perform as intended in cases of clinical dysfunction that impairs VOR (12). In ongoing work, our group is developing algorithms to detect VOR.

The example angular displacement results in Figure 6 show a trend of the EOGbased estimates being less than the video-based estimates. This bias was consistent across trials, leading us to believe that the dominant source of error is unmodeled structure rather than sensor noise. For example, our model does not explicitly account for the possibility of VOR induced by translation (rather than rotation) of the head, nor the offset between the eye and the center of head rotation. Instead, it assumes that they are both zero (or equivalently, that the gaze target point is infinitely far away), and encodes the inaccuracy of that assumption in the covariance *C_ω_*.

There is also the possibility that some of the error is due to the compared videobased eye tracker itself. Though the Tobii system advertises an accuracy of 0.3°, independent investigations report a less ideal result of 2.46° when chin position and lighting are not tuned for maximum performance (27; 6). Since the video eye tracking data was defined as “truth” in our validation study, any error introduced from the video eye tracker itself was incorrectly attributed to the model’s performance.

Moreover, there is a difference between the accuracies of desktop and mobile video eye trackers. For example, the HTC Vive Pro Eye (HTC Corporation, Taoyuan, Taiwan) was independently reported to have an accuracy of 4.16° - 4.75° (25), and the Microsoft Hololens 2 is reported to have 1.5° - 3° (22). The current methodology is focused on free-living (i.e., mobile) eye tracking and calibration. Rather than compare the accuracy of the model’s performance to goldstandard desktop systems, the comparison to mobile (e.g., VR system) eye trackers is more appropriate. Thus, an error of 3.54° would be par for mobile eye trackers.

## Conclusion

To calibrate EOG signals in free-living without needing a reference video eye tracker, we have developed a model that relates EOG and head movement signals under the assumption of VOR. This model is ideal for inference via extended Kalman filtering. Doing so yields simultaneous and continual estimation of gaze direction and the EOG calibration coefficients. The quality of these estimates was validated by computing angular displacements over a pattern of saccades and comparing them to those computed by a video-based eye tracker. On average, the two approaches differed by 3.54°. Our methodology enables mobile gaze estimation with just an EOG and gyroscope, poised for long-duration use in free-living.

## Ethics and Conflict of Interest

The author(s) declare(s) that the contents of the article are in agreement with the ethics described in http://biblio.unibe.ch/portale/elibrary/BOP/jemr/ethics. html and that there is no conflict of interest regarding the publication of this paper.

## Disclaimer

DISTRIBUTION STATEMENT A. Approved for public release. Distribution is unlimited. This material is based upon work supported by the Department of the Army under Air Force Contract No. FA8702-15-D-0001. Any opinions, findings, conclusions or recommendations expressed in this material are those of the author(s) and do not necessarily reflect the views of the Department of Defense, the U.S. Army, or the U.S. Army Medical Materiel Development Activity.
